# *De novo* assembly and comparative transcriptome analysis of *Euglena gracilis* in response to anaerobic conditions

**DOI:** 10.1186/s12864-016-2540-6

**Published:** 2016-03-03

**Authors:** Yuta Yoshida, Takuya Tomiyama, Takanori Maruta, Masaru Tomita, Takahiro Ishikawa, Kazuharu Arakawa

**Affiliations:** Institute for Advanced Biosciences, Keio University, Tsuruoka, Yamagata 997-0052 Japan; Systems Biology Program, Graduate School of Media and Governance, Keio University, Fujisawa, Kanagawa 252-0882 Japan; Department of Life Science and Biotechnology, Faculty of Life and Environmental Science, Shimane University, 1060 Nishikawatsu, Matsue, Shimane 690-8504 Japan; Core Research for Evolutional Science and Technology (CREST), Japan Science and Technology Agency (JST), Chiyoda-ku, Tokyo, 102-0076 Japan

**Keywords:** *Euglena gracilis*, Wax ester fermentation, Transcriptome analysis

## Abstract

**Background:**

The phytoflagellated protozoan, *Euglena gracilis*, has been proposed as an attractive feedstock for the accumulation of valuable compounds such as β-1,3-glucan, also known as paramylon, and wax esters. The production of wax esters proceeds under anaerobic conditions, designated as wax ester fermentation. In spite of the importance and usefulness of *Euglena*, the genome and transcriptome data are currently unavailable, though another research group has recently published *E.gracilis* transcriptome study during our submission. We herein performed an RNA-Seq analysis to provide a comprehensive sequence resource and some insights into the regulation of genes including wax ester metabolism by comparative transcriptome analysis of *E.gracilis* under aerobic and anaerobic conditions.

**Results:**

The *E.gracilis* transcriptome analysis was performed using the Illumina platform and yielded 90.3 million reads after the filtering steps. A total of 49,826 components were assembled and identified as a reference sequence of *E.gracilis*, of which 26,479 sequences were considered to be potentially expressed (having FPKM value of greater than 1). Approximately half of all components were estimated to be regulated in a *trans*-splicing manner, with the addition of protruding spliced leader sequences. Nearly 40 % of 26,479 sequences were annotated by similarity to Swiss-Prot database using the BLASTX program. A total of 2080 transcripts were identified as differentially expressed genes (DEGs) in response to anaerobic treatment for 24 h. A comprehensive pathway enrichment analysis using the KEGG pathway revealed that the majority of DEGs were involved in photosynthesis, nucleotide metabolism, oxidative phosphorylation, fatty acid metabolism. We successfully identified a candidate gene set of paramylon and wax esters, including novel β-1,3-glucan and wax ester synthases. A comparative expression analysis of aerobic- and anaerobic-treated *E.gracilis* cells indicated that gene expression changes in these components were not extensive or dynamic during the anaerobic treatment.

**Conclusion:**

The RNA-Seq analysis provided comprehensive transcriptome information on *E.gracilis* for the first time, and this information will advance our understanding of this unique organism. The comprehensive analysis indicated that paramylon and wax ester metabolic pathways are regulated at post-transcriptional rather than the transcriptional level in response to anaerobic conditions.

**Electronic supplementary material:**

The online version of this article (doi:10.1186/s12864-016-2540-6) contains supplementary material, which is available to authorized users.

## Background

Microalgae have recently attracted interest as a renewable source of biofuel and valuable compounds such as carotenoids, long chain unsaturated fatty acids, pigments, and polysaccharides [1, 2]. Microalgae are considered to have advantages over plants currently utilized for energy feedstock due to their fast growth rates, high lipid productivity, and cultivation on non-arable land areas that not compete with food production [3].

*Euglena gracilis* is a unicellular phytoflagellate that is widely distributed in freshwater, and has been proposed as a feedstock to produce biodiesel and various valuable compounds. For example, *E. gracilis* has been shown to accumulate a storage polysaccharide, a β-1,3-glucan known as paramylon, under aerobic conditions. Under optimal culture conditions *E. gracilis* has the ability to accumulate up to 50 % of paramylon per dry weight of the cells [4]. Paramylon has potential applications not only in biomedical field due to its immunomodulation and anti-tumor activities, but also in industrial materials such as nanofibers [5, 6].

Paramylon is an important starting material for wax ester production under anaerobic conditions [7, 8]. When aerobically grown *E. gracilis* cells are transferred into anaerobic conditions, they degrade paramylon to actively synthesize and accumulate wax esters, which consist of medium-chain fatty acids and alcohols including 14:0 carbon chains as the major constituent. This phenomenon is designated wax ester fermentation due to the concomitant generation of ATP without any energy loss during wax ester production [7]. Due to its low freezing point with a good Cetane number (66.2) [9], myristic acid (C14:0) has greater potential as a drop-in jet fuel than other algae produced medium-length fatty acids such as palmitic acid (C16:0) and stearic acid (C18:0).

The wax ester fermentation pathway proceeds across cellular compartments; glycolysis occurs in the cytoplasm, fatty acid biosynthesis in the mitochondria, and wax ester synthesis in the microsoms. The metabolic enzymes related to this pathway have biochemically analyzed. Anaerobic *de novo* fatty acid synthesis in mitochondria has been shown to utilize acetyl-CoA as a primer and a C2 donor, stemming from pyruvate *via* oxygen-labile pyruvate:NADP^+^ oxidoreductase instead of an ordinary pyruvate dehydrogenase complex [10]. The pathway then proceeds *via* reversible enzymatic steps of β-oxidation by participation of a medium-chain *tran*-2-enoyl CoA reductase instead of acyl CoA dehydrogenase [11]. However, the molecular details of most metabolic enzymes and the regulatory mechanism of wax ester fermentation in response to aerobic or anaerobic conditions remain largely unknown.

In addition to its advantage as a biofuel feedstock, *E. gracilis* is rich in nutrients such as vitamins, minerals, and well-balanced amino acids [12, 13]; therefore, it is used as a source of dietary supplement, in manufacture of cosmetics, and in the fortification of livestock feed. After the production of biodiesel by *E. gracilis*, the residual biomass can be converted into valuable industrial materials. Thus, *E. gracilis* is an ideal microalgal species for biodiesel and biomass production.

In spite of increasing interest in *Euglena*, genome and transcriptome data are not currently available, except for the chloroplast genome and a limited number of EST analyses [14–16]. An RNA-Seq analysis is one of the more superior molecular techniques being extensively used for the comprehensive gene expression profiling of living organisms including non-model organisms for which genomic information is currently limited, such as *E.gracilis*. In the present study, we performed RNA-Seq analysis to annotate functional transcripts and provide a comprehensive sequence resource for *E.gracilis*. Moreover, a comparative transcriptome analysis of *E.gracilis* under aerobic and anaerobic conditions provided some insights into the regulation of wax ester metabolism.

## Results and discussion

### RNA-Seq and *de novo* assembly

In order to obtain a reference sequence of expressed genes in *E.gracilis*, we constructed a RNA-Seq library from mRNA, isolated from photoheterotrophically grown cells. In total, 98.5 million raw reads (9.85 Gb) were obtained using Illumina HiSeq2000. After trimming and removing low quality reads, 90.3 million clean reads (9.03 Gb) were obtained. Detailed information on the assembly is shown in Table 1. In order to obtain a comprehensive sequence set for *E.gracilis* transcriptome, all clean reads were assembled together using the Trinity program [17]. We obtained a total of 113,295 transcripts, including putative splice variants. The transcripts had a mean length of 845 bp and N50 of 1604 bp. Among all transcripts, the Trinity program suggested that 49,826 sequences were unique components. During our submission, O’Neill et al. [18] published a transcriptome study of *E.gracilis*. The assembled number of sequence was almost the same as ours. A previous study indicated that transcripts with low Fragments Per Kilobase of exon per Million fragments mapped (FPKM) were assembly errors [19]; therefore, in the present study, 49,754 and 26,479 sequences with FPKM >1 were designated as “potentially expressed” transcripts and components, respectively. The assembly data obtained are valuable for contributing to genome annotation in future studies and the identification of novel genes in this unique organism. One of the unique characteristics of *E.gracilis* transcripts is the presence of a spliced (or short)-leader (SL) sequence at their 5′-end in a *trans*-splicing manner [20]. Within our set of 26,479 potentially expressed components, we estimated the number of transcripts that attached the protruding SL-sequence. The *E.gracilis* SL-sequence was shown to consist of 26 nucleotides after processing of precursor 5S rRNA. These SL-sequences had slight variations, especially in the leader sequence [21]. Therefore, we selected 12 conserved nucleotides (TATTTTTTTTCG) from its 3′-terminus as a query sequence, and found that 53.6 % of components included the SL-sequence, which was consistent with previous findings in which approximately 60 % of all *in vitro* translations were suppressed by a treatment with a complementary oligonucleotide against *E.gracilis* SL-sequence [20].

**Table 1:** Summary of *E.gracilis* RNA-Seq data

Read data (bps)	9.03G
Total transcripts (seq)	113,295
FPKM≧1 out of total transcripts (seq)	49,754
Components (seq)	49,826
FPKM≧1 out of components (seq)	26,479
Median (nucl)	845
Contig N50 (nucl)	1604
Max length (nucl)	21,550
GC contents (%)	63.17
Components with SL-sequence^a^	14,186/26,479 (53.6 %)

^a^Counted sequences from the representing potentially expressed components (26,479 seq) with conserved 12 nucleotides (TATTTTTTTTCG)

### Annotation of *Euglena* transcripts

**Fig. 1:**
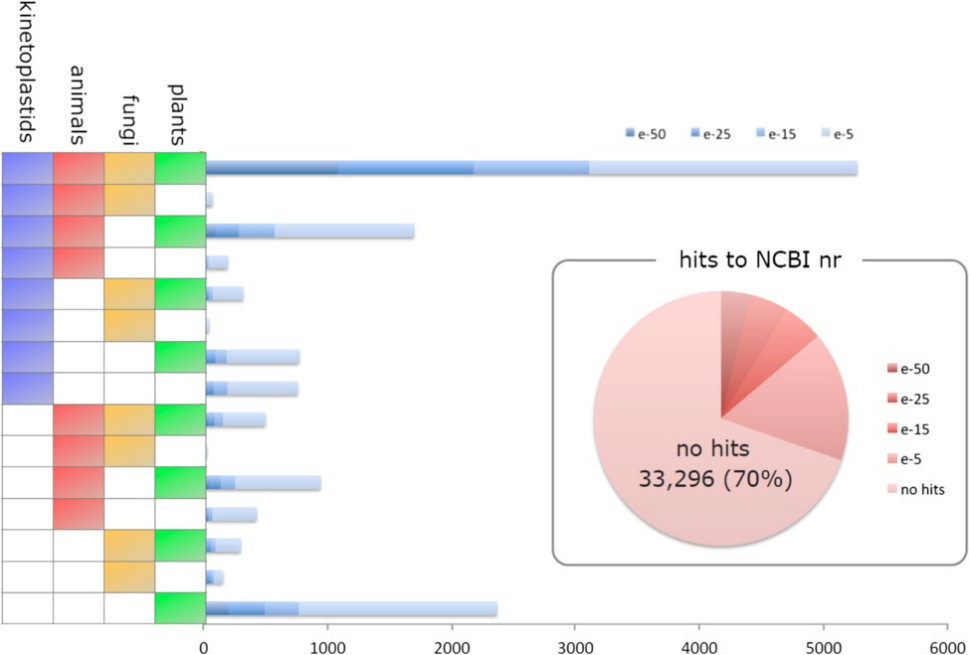
Taxonomic distribution of annotated components by BLASTX search against genomic databases of plants, animals, fungi, and kinetoplastids. The genomic databases used are listed in Methods section. Significant matches were defined as those with e-values < 1e^−5^. The pie chart shows the percentage of no hits category of the BLAST results to NCBI nr database. The bar chart was added to visualize  gene numbers belonging to each category

Of the 26,479 components, 11,314 sequences (42.7 %) showed significant similarity to known sequences in the Swiss-Prot database using the BLASTX program with an E-value cut-off of 1e^−5^. This proportion of similarity implies that approximately 60 % of our set of components are genes with unknown function. To compare a similarity category of *Euglena* components with those of other organisms, the components were further subjected to BLASTX search against putative proteins (e-value < 1e^−10^) from plants, fungi, animals, and kinetoplstids genome databases. As shown in Fig. 1, though the majority of components (5,270; 38.2 %) were belonging to all categories, significant number of components (757; 5.5 %) had homology to Kinetoplastida only. These Kinetoplastid sequences corresponded to unique proteins including trypanothione reductase, Kinetoplastid membrane protein 11, kinesin-like protein K39, cruzipain, and others, as expected. Some components had homology to only plant (2,366; 17.1 %) or animal (452; 3.1 %) categories, reflecting that data were obtained from cells grown under photoheterotrophic conditions. Overall the assigned components were widely distributed in all categories, supporting previous findings suggesting that the *Euglena* genome was a hybrid of photoautotrophic and heterotrophic genomes because of endosymbiotic gene transfer events [16]. A complete genomic sequence analysis will lead to a deeper understand of the evolutionary events that occurred in *Euglena*.

**Fig. 2:**
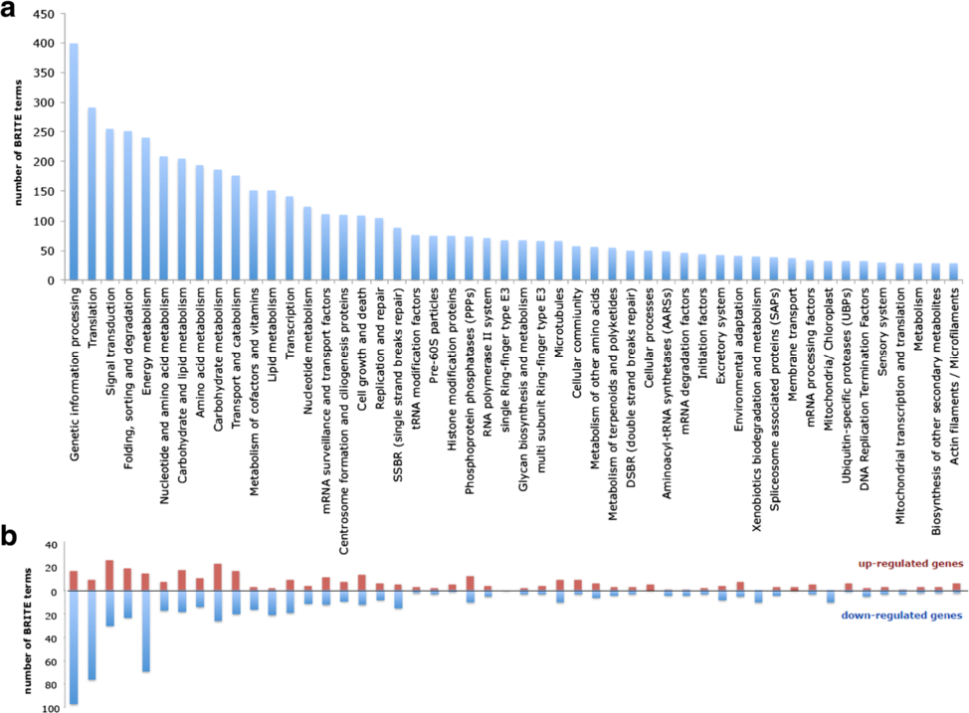
KEGG pathway analysis of assembled components in *E.gracilis*. The data exhibited are significantly enriched in the top 50 categories classified by KEGG BRITE hierarchy level 2 (http://www.genome.jp/kegg/kegg3b.html). **a** Illustration of the counts of the BRITE terms in all assembled components. **b** Analysis only for 800 up-regulated and 1280 down-regulated DEGs identified with FDR <0.05 in the comparison of aerobic and anaerobic states. The red and blue bars indicate the number of BRITE terms in up- and down-regulated genes, respectively

A pathway analysis based on KEGG BRITE hierarchy level 2 was performed to understand the functional categories of *E.gracilis* transcripts (Fig. 2a). Highly represented pathways included ‘Genetic information processing (399 components)’, ‘Translation (291 components)’, and ‘Energy metabolism (239 components)’, indicating active status of photoheterotrophically grown *E.gracilis* cells. It is worth noting that when the analysis is just focusing on differentially expressed genes (DEGs), majority of genes belonging to these categories were down-regulated in response to anaerobic condition (Fig. 2b). Later, we provide a detailed discussion on this phenomenon.

### Paramylon and wax ester metabolic pathway

Paramylon and wax esters are storage compounds in *E.gracilis* under aerobic and anaerobic conditions, respectively. Paramylon is synthesized from UDP-glucose *via* paramylon synthase (β-1,3-glucan synthase or callose synthase) [22]. Based on its similarity to other β-1,3-glucan synthases, two components (comp36539_c1_seq4 and comp36157_c0_seq1) may be involved in the synthesis of paramylon (Additional file 1: Table S1). By comparing both FPKM values, the comp36157_c0_seq1 protein appears to be responsible for the synthesis due to its higher expression level than comp36539_c1_seq4. Previous studies reported that glucanase and phosphorylase were both involved in the degradation of paramylon [23, 24]. Seven components were annotated as β-1,3-glucanase; three of these components were recently purified from *Euglena* extract and identified as endo-form glucanase [25]. On the other hand, no components with significant similarity to 1,3-beta-D-glucan phosphorylase were found in our assembled database.

**Fig. 3:**
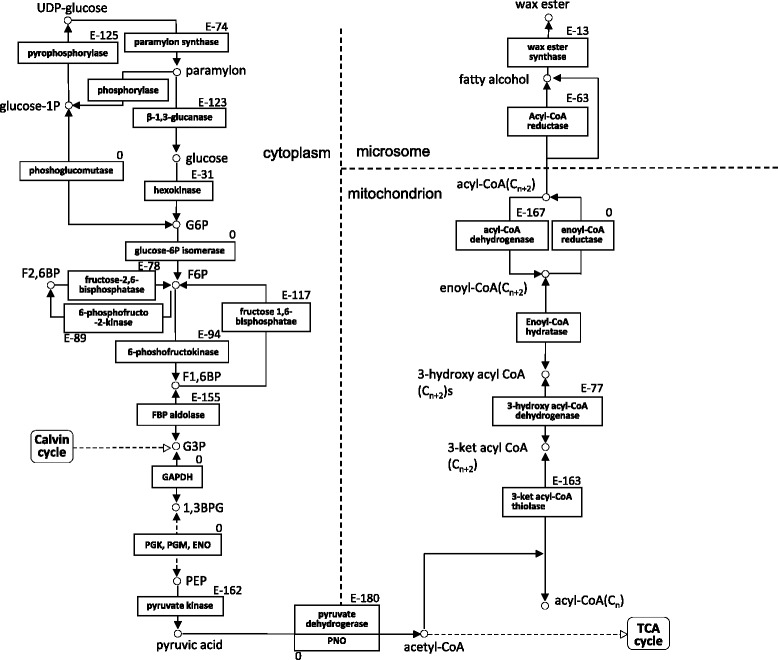
Pathway map for paramylon and wax ester metabolism in *E.gracilis*. The enzymes involved in these pathways are provided. G6P, glucose 6-phosphate; F6P, fructose 6-phosphate; F1,6BP, fructose-1,6-bisphosphate; F2,6BP, fructose-2,6-bisphosphate; G3P, glyceraldehyde 3-phosphate; GAPDH, glyceraldehyde 3-phosphate dehydrogenase; 1,3BPG, 1,3-bisphosphoglycerate; PGK, phosphoglycerate kinase; PGM, phosphoglycerate mutase; ENO, enolase; PEP, phosphoenolpyruvate; PNO, pyruvate:NADP^+^ oxidoreductase. The best e-values for individual genes are indicated in the top right corner of boxes shown enzyme names

Wax esters are synthesized from the degradation of paramylon *via* glycolysis in the cytosol, and a mitochondrial *de novo* fatty acid synthesis pathway under anaerobic conditions [7, 8] (Fig. 3). All components with functional annotations associated with the pathway for wax ester fermentation were identified, as shown in Additional file 1: Table S1. All components, some of which contained several isoforms, were annotated in the central glycolytic or gluconeogenesis pathway. One component was identified as cytosolic fructose-1,6-bisphosphatase, which is thought to play an intermediate role between glycolysis and gluconeogenesis [26]. In *Euglena*, the conversion of pyruvate to acetyl-CoA occurs *via* a unique oxygen-sensitive pyruvate:NADP^+^ oxidoreductase and pyruvate dehydrogenase complex [10, 27], both of which were identified in the assembly data. Under anaerobic conditions, *de novo* fatty acid synthesis in mitochondria proceeds *via* the reversible enzymatic steps of β-oxidation through the participation of a *tran*-2-enoyl CoA reductase instead of an acyl CoA dehydrogenase [9]. Sixteen components involved in mitochondrial *de novo* fatty acid synthesis were annotated. Six of these components were for 3-ketacyl CoA thiolase [28], 3 were for 3-hydroxy acyl CoA dehydrogenase, 2 were for enoyl CoA hydratase, 3 were for acyl CoA dehydrogenase, and 2 were for *tran*-2-enoyl CoA reductase. We identified 6 novel components that included both wax ester and triacylglycerol synthesis [30]; these components are annotated as wax ester synthase/acyl-CoA:diacylglycerol acyltransferase (WSD). In addition, we identified a component for wax ester synthase, corresponding to the cDNA reported by Teerawanichpan and Qiu [29]. We are greatly interested in whether or not the *E. gracilis* WSD family actually synthesizes wax ester. Consequently, our literature search focused heavily on genes that may participate in paramylon and wax ester metabolism. Further, biochemical and genetic analyses are necessary to characterize the function of these *E. gracilis* genes.

### Comparison of differentially expressed genes between *E.gracilis* cells in response to anaerobic conditions

**Fig. 4:**
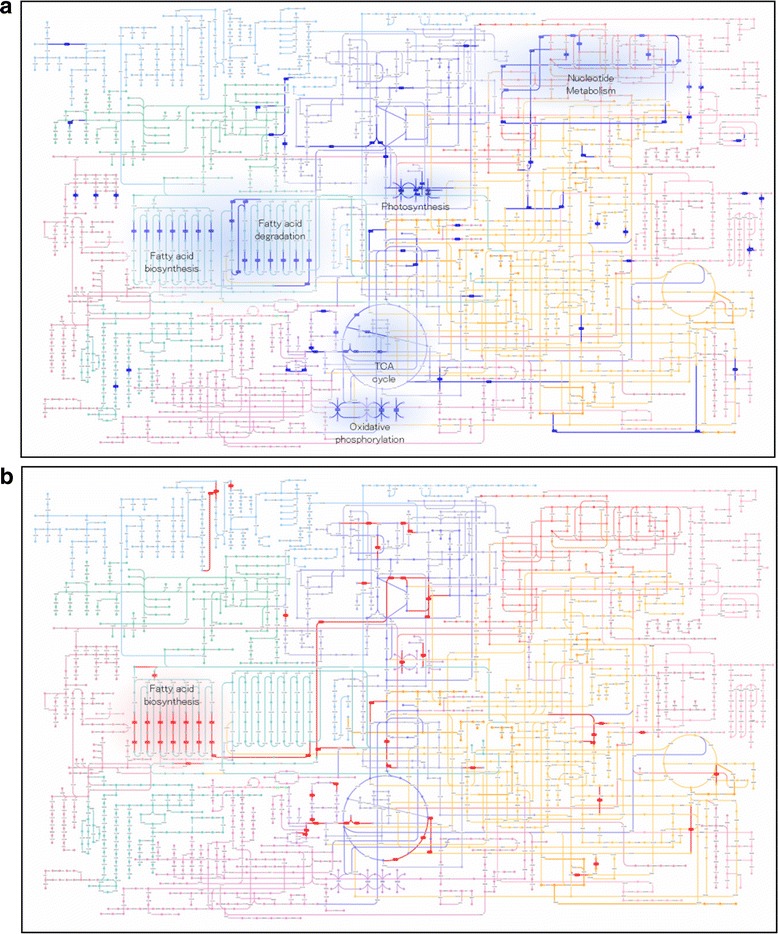
Representation of pathway maps for differential expressed genes in response to anaerobic treatment for 24 h. Given are values for FPKM ratios corresponding to components above the 2-fold change threshold. Blue (**a**) and red (**b**) colors highlight down- and up-regulated genes, respectively. Some genes related to pathways involving photosynthesis, nucleotide metabolism, TCA cycle, oxidative phosphorylation, fatty acid degradation, and a part of fatty acid biosynthesis showed lower expression in response to anaerobic treatment for 24 h. Zoomable interactive maps generated using Pathway Projector [39] are available online at: [Down-regulated pathways] http://ws.g-language.org/g4/main.cgi?diaAtabareaname=1&flag=00001, [Up-regulated pathways] http://ws.g-language.org/g4/main.cgi?diaAtabareaname=1&flag=00002

We performed a comparative transcriptome analysis to elucidate the molecular mechanisms that enable *E.gracilis* cells, under anaerobic conditions, to activate the degradation of paramylon and produce wax esters. cDNA libraries were prepared from wild-type strain Z cells, shaded for 12 or 24 h to avoid oxygenic photosynthesis and archive anaerobic conditions. Sequencing of the individual cDNA libraries (*n* = 3) with an Illumina MiSeq yielded 15.8 M - 24.9 M reads (Additional file 2: Table S2). Approximately 95 % of all reads were finally mapped to the reference transcripts described above. Genes with triplicate expression profiles implying a false discovery rate (FDR) <0.05 were identified as statistically significant differentially expressed genes (DEGs). A total of 2080 transcripts were identified as DEGs. Of these, 800 and 1280 transcripts were significantly up- and down-regulated in cells treated anaerobically for 24 h, respectively (Additional file 3). One hundred and sixty-two transcripts for these DEGs contained multiple putative isoforms (having the same component IDs and different seq numbers in Trinity) that showed significant expression differences, indicating the presence of splice variants. However, a close inspection of individual possibilities does not suggest that alternative splicing plays a significant role in the transcriptional regulation resulting from anaerobic treatment of *E. gracilis* (data not shown). Furthermore, by employing a stringent cut-off of above 2-fold change in gene expression, a subset of 293 genes was modulated in response to anaerobic conditions for 24 h (Additional file 3). In order to analyze the metabolic pathways significantly altered under anaerobic conditions, we conducted a KEGG pathway enrichment analysis. Among the 800 up- and 1280 down-regulated genes, 324 and 448 genes were mapped to KEGG ID (KO_ID) by KEGG KAAS, respectively. These genes were enriched in several KEGG pathways (Additional file 3). As shown in Fig. 4, although a limited number of pathways exhibited significant alterations, pathways termed “photosynthesis”, “nucleotide metabolism”, “TCA cycle”, and “oxidative phosphorylation” significantly declined after anaerobic treatment. To generate ATP under anaerobic conditions, it has been suggested that *E.gracilis* utilizes fumarate as the terminal acceptor of electron flux instead of O_2_; this hypothesis is based on the presence of rhodoquinone-9 in *E.gracilis*, which is an essential component in fumarate reduction in mitochondria during anaerobic adaptation [31]. Thus down-regulation of aerobic metabolic pathways would be reasonable. It is also reasonable that a series of genes involved in photosynthesis processes were markedly down-regulated because of shading during anaerobic treatment for 24 h.

The pathway related to fatty acid metabolism was altered in response to anaerobic conditions, and components of this pathway, such as comp36489_c0_seq1 (annotated as fatty acid synthase subunit beta), were not involved in wax ester metabolic pathway. Suppression of several gene categories mentioned above was also confirmed by GO enrichment analysis. No significantly enriched GO terms were found for up-regulated genes, but down-regulated genes contained six terms, including translation, macromolecule biosynthetic process, and photosynthesis, as listed in Table 2. Matsuda et al. [32] used a comparative metabolic profiling analysis to demonstrate that many metabolites, including nucleotide cofactors and amino acids, were markedly altered in response to changes in aeration conditions. Thus the results obtained in the present study were consistent with previous findings on metabolome profiling.

**Table 2:** Enriched GO terms of down-regulated genes in anaerobic state

GO ID	GO annotation	q-value
GO:0006412	translation	1.56E-11
GO:0009059	macromolecule biosynthetic process	2.78E-09
GO:0015979	photosynthesis	6.28E-09
GO:0006091	generation of precursor metabolites and energy	6.12E-06
GO:0006118	obsolete electron transport	0.00073
GO:0006414	translational elongation	0.00848

Regarding expression changes in genes related to paramylon and wax ester metabolism, as described above, absolute fold-changes were less than 1.7-fold after 24 h anaerobic treatment (Additional file 1: Table S1). Furthermore, there was no consistency in most gene expression levels. For example, the expression levels for pyruvate:NADP^+^ oxidoreductase (PNO), which is considered to be a key regulatory enzyme for wax ester production due to its oxygen sensitivity [10], and for *tran*-2-enoyl CoA reductase 1 (TER1), were down-regulated even though we expected them to be up-regulated. Thus, when viewed globally, the gene expression changes that occurred during anaerobic treatment were not extensive or dynamic (Additional file 4). These limited expression changes do not appear to play a critical role in the activation of paramylon degradation and wax ester production under anaerobic conditions. In contrast to *E.gracilis*, a previous study reported that, in the diatom *Phaeodactylum tricornutum*, the transcriptional levels of some genes associated with glucan and fatty acid metabolism were altered in response to light/dark cycles, indicating significance of transcriptional regulation of central metabolic pathways [33]. On the other hand, post-translational modifications, such as phosphorylation and acetylation, are known to play key roles in switching between respiratory and fermentative metabolism in response to aerobic and anaerobic conditions in yeast cells [34]. Proteomic characterizations of post-translational modifications such as phosphorylation may provide important insights into the regulatory mechanism of wax ester fermentation in *E.gracilis* responding to changing oxygen conditions.

## Conclusion

*E.gracilis* has atypical metabolic processes that provide extensive capacities for adaptation to extreme environmental conditions. One such process is a unique wax ester fermentation pathway activated under anaerobic conditions [7]. In the present study, a *de novo* transcriptome analysis of *E.gracilis* was performed for the first time in an attempt to obtain a mechanistic understanding of anaerobic wax ester fermentation. The assembled and annotated sequence data identified the existing genes available from reference sequences and will benefit the *Euglena* research community. A comparative expression analysis showed that DEGs associated with energy metabolism, such as TCA cycle and oxidative phosphorylation, were altered in response to anaerobic conditions. Furthermore, this study obtained sequence information regarding enzymes potentially responsible for paramylon and wax ester metabolism. These sequences enabled us not only to elucidate the regulation mechanism of wax ester fermentation in response to changes in aeration conditions, but also to facilitate *Euglena* research by addressing basic biochemical, physiological, and evolutionary studies.

## Methods

### *Euglena* strain and culture

The wild-type *E.gracilis* strain Z was grown photoheterotrophically in Koren-Hutner (KH) medium [35] on a rotary shaker (120 rpm) under continuous light (100 μmol photons m^−2^ s^−1^) at 26 °C. Cell numbers were measured using the CASY Cell Counter and Analyzer System (Roche Applied Science, Basel, Switzerland). Stationary phase cells were incubated under anaerobic conditions in a sealed and shaded environment after N_2_-gas sparging for 5 min. The anaerobic state of cultures during experiments was confirmed by measuring oxygen concentration using an oxygen sensor (Fibox3, Taitec, Japan).

### RNA extraction

Total RNA was extracted from 1 mL of cultures (>10^6^ cells) using RNAiso reagent (Takara, Japan), and purified using RNeasy Plus Universal Kits (Qiagen) following the manufacturer’s instructions. RNA quality and quantity were evaluated using Bioanalyzer 2100 (Agilent, CA).

### RNA-Seq library preparation and sequencing

In order to obtain the *Euglena* reference sequence, an RNA-Seq library was constructed following the procedure recommended by Illumina. Briefly, mRNA was purified from 4 μg of the total RNA sample extracted from *E.gracilis* strain Z at stationary phase. It was then fragmented and converted to double-stranded DNA using TruSeq RNA Sample Preparation Kit v2 (Illumina). After a quality assessment and quantification of the sample libraries by Bioanalyzer 2100 (Agilent, CA) and a KAPA library quantification kit (KAPA Biosystems, UK), the library was sequenced using an Illumina HiSeq 2000 at the Exeter Sequencing Service at Exeter University. Next, sequencing for a detailed comparative gene expression analysis was performed using Illumina MiSeq after preparation of the RNA-Seq library from aerobic- and anaerobic-treated cells, following the same procedures as described above.

### *De novo* transcriptome assembly

To obtain high-quality clean reads, the raw reads were filtered to remove reads with adaptor sequences, low-quality reads (Phred quality score <20 bp), and reads with a high percentage of unidentified nucleotides, using Perl script with G-language Genome Analysis Environment [36]. *De novo* assembly of the clean reads was carried out using Trinity software (version: trinity/r2014-04-13p1) with default parameters and no reference sequence. The sequences resulting from the *de novo* Trinity assembly were called unigenes. In order to annotate unigenes, a BLASTX search against the UniProt database was conducted with an E-value cut-off of 1e^−5^. The following genomic databases were used for the taxonomic distribution of annotated components : plants (*Chlamydomonas reinhardtii*, http://www.ncbi.nlm.nih.gov/pubmed /17932292; *Arabidopsis thaliana*, https://www.arabidopsis.org/); animals (*Drosophila melanogaster*, http://flybase.org/; *Caenorhabditis elegans*, https://www.wormbase.org/); fungi (*Saccharomyces cerevisiae*, http://www.ensembl.org/index.html); kinetoplastids (*Trypanosoma cruzi*, *Trypanosoma brucei*, *Leishmania major*, http://www.ncbi.nlm.nih.gov/). A venn diagram was drawn using Venny program (http://bioinfogp.cnb.csic.es/tools/venny/). The Blast2GO program [37] and Kyoto Encyclopedia of Genes and Genomes (KEGG) database [38] (http://www.genome.jp/kegg/) were used to identify the Gene ontology (GO) annotation and biological pathways in *E.gracilis*, respectively. Results of pathway enrichment were visualized using Pathway Projector [39].

### Analysis of differentially expressed genes (DEG) in anaerbiosis *E.gracilis* cells

A differential gene expression analysis was performed using edgeR [41] (with FDR <0.05) on the reads that mapped with the bowtie2 software [40] to the unigenes assembled as described above For expression abundance estimation, the FPKM (Fragments Per Kilobase of exon per Million mapped fragments) values were computed using RSEM software [42] (http://www.ncbi.nlm.nih.gov/pubmed/21816040). Fold changes in differentially expressed genes in anaerobic-treated *E.gracilis* cells were calculated using the log_2_ ratio of FPKM. Based on the FPKM values, the pathway maps were generated using Pathway Projector. GO terms were extracted by matching UniProt-GOA associations with BLASTX search results (e-values <1e^−5^) and analyzed for statistically significant enrichment using GOstat for Biological Process terms (depth = 3; q-value ≤ 0.01; Benjamini correction for FDR) [43].

### Availability of supporting data

The sequencing and transcriptome assembly data sets supporting the results of this study are deposited and available at the DDBJ Sequence Read Archive (DRA) with accession number SRP060591 and GDJR00000000.1 (GenBank), respectively. The data set supporting the results of this article is included within the article.

## Additional files

Additional file 1: Table S1. Summary of the homology search by BLASTX program for the selected components related to paramylon and wax ester metabolism in *E.gracilis*. The localization of individual deduced proteins is predicted by using TargetP 1.1 program (http://www.cbs.dtu.dk/services/TargetP/) and/or TMHMM v.2.0 (http://www.cbs.dtu.dk/services/TMHMM/). (DOCX 47 kb) Additional file 2: Table S2. Summary of reads for sequencing of individual cDNA libraries constructed from *E.gracilis* treated with aerobic- and anaerobic conditions. (DOCX 18 kb) Additional file 3: Data and list for 2080 DEGs in response to anaerobic conditions. (XLS 1336 kb) Additional file 4: Figure S1. Hierarchical clustering analysis for 2080 DEGs in response to anaerobic conditions. Each row and column represent individual DEGs and time points, respectively. For each time point, the experiment was performed three times each. (PPTX 91 kb)

## References

[1] Mata TM, Martins AA, Caetano NS (2010). Microalgae for biodiesel production and other applications: A review. Renew Sustain Energy Rev.

[2] Markou G, Nerantzis E (2013). Microalgae for high-value compounds and biofuels production: a review with focus on cultivation under stress conditions. Biotechnol Adv.

[3] Stephens E, Ross IL, King Z, Mussgnug JH, Kruse O, Posten C (2010). An economic and technical evaluation of microalgal biofuels. Nature Biotech.

[4] Barras DR, Stone BA. Carbohydrate composition and metabolism in *Euglena*. In: Edited by Buetow DE. The Biology of Euglena vol. 2. Academic Press, New York; 1968. 149–191.

[5] Watanabe T, Shimada R, Matsuyama A, Yuasa M, Sawamura H, Yoshida E (2013). Antitumor activity of the β-glucan paramylon from *Euglena* against preneoplastic colonic aberrant crypt foci in mice. Food Funct.

[6] Shibakami M, Tsubouchi G, Nakamura M, Hayashi M (2013). Preparation of carboxylic acid-bearing polysaccharide nanofiber made from euglenoid β-1,3-glucans. Carbohydr Polym.

[7] Inui H, Miyatake K, Nakano Y, Kitaoka S (1982). Wax ester fermentation in *Euglena gracilis*. FEBS Lett.

[8] Inui H, Miyatake K, Nakano Y, Kitaoka S (1984). Fatty acid synthesis in mitochondria of *Euglena gracilis*. Eur J Biochem.

[9] Yanowitz, J, Ratcliff, MA, McCormick, RL, Taylor JD, Murphy, MJ. Compendium of experimental cetane numbers. In: Technical Repot. National Renewable Energy Laboratory. 2014. http://www.nrel.gov/docs/fy14osti/61693.pdf

[10] Inui H, Miyatake K, Nakano Y, Kitaoka S (1990). Pyruvate:NADP^+^ oxidoreductase from *Euglena gracilis*: mechanism of O_2_-inactivation of the enzyme and its stability in the aerobe. Arch Biochem Biophys.

[11] Hoffmeister M, Piotrowski M, Nowitzki U, Martin W (2005). Mitochondrial *trans*-2-enoyl-CoA reductase of wax ester fermentation from *Euglena gracilis* defines a new family of enzymes involved in lipid synthesis. J Biol Chem.

[12] Kott Y, Wachs AM (1964). Amino acid composition of bulk protein of *Euglena*  grown in waste water. Appl Microbiol.

[13] Baker ER, McLaughlin JJA, Hutner SH, DeAngelis B, Feingold S, Frank O (1981). Water-soluble vitamins in cells and spent culture supernatants of Poteriochromonas stipitata, *Euglena gracilis*, and *Tetrahymena thermophila*. Arch Microbiol.

[14] Hallick RB, Hong L, Drager RG, Favreau MR, Monfort A, Orsat B (1993). Complete sequence of *Euglena gracilis* chloroplast DNA. Nucleic Acids Res.

[15] Durnford DG, Gray MW (2006). Analysis of *Euglena gracilis* plastid-targeted proteins reveals different classes of transit sequences. Eukaryot Cell.

[16] Ahmadinejad N, Dagan T, Martin W (2007). Genome history in the symbiotic hybrid *Euglena gracilis*. Gene.

[17] Grabherr MG, Haas BJ, Yassour M, Levin JZ, Thompson DA, Amit I (2011). Full-length transcriptome assembly from RNA-Seq data without a reference genome. Nat Biotechnol.

[18] Zhao J, Ohsumi TK, Kung JT, Ogawa Y, Grau DJ, Sarma K (2010). Genome-wide identification of polycomb-associated RNAs by RIP-seq. Mol Cell.

[19] Tessier LH, Keller M, Chan RL, Fournier R, Weil JH, Imbault P (1991). Short leader sequences may be transferred from small RNAs to pre-mature mRNAs by *trans*-splicing in *Euglena*. EMBO J.

[20] Frantz C, Ebel C, Paulus F, Imbault P (2000). Characterization of *trans*-splicing in Euglenoids. Curr Genet.

[21] O’Neill EC, Trick M, Hill L, Rejzek M, Dusi RG, Hamilton CJ (2015). The transcriptome of *Euglena gracilis* reveals unexpected metabolic capabilities for carbohydrate and natural product biochemistry. Mol Biosyst.

[22] Marechal LR, Goldemberg SH (1964). Uridine diphosphate glucose-β-1,3-glucan β-3-glucosyltransferase from *Euglena gracilis*. J Biol Chem.

[23] Barras DR, Stone BA (1969). Beta-1,3-glucan hydrolases from *Euglena gracilis*. I. The nature of the hydrolases. Biochim Biophys Acta.

[24] Goldemberg SH, Marechal LR. Laminaribiose phosphorylase and β-1,3-oligoglucan phosphorylase from *Euglena*. In: Edited by Colowick SP, Kaplan NO. Method in Enzymology vol. 28. Academic Press; 1972. 953–960.

[25] Takeda T, Nakano Y, Takahashi M, Konno N, Sakamoto Y, Arashida R (2015). Identification and enzymatic characterization of an endo-1,3-beta-glucanase from *Euglena gracilis*. Phytochemistry.

[26] Ogawa T, Kimura A, Sakuyama H, Tamoi M, Ishikawa T, Shigeoka S. Identification and characterization of cytosolic fructose-1,6-bisphosphatase in *Euglena gracilis. Biosci. Biotechnol. Biochem*. 2015, in press. Doi:10.1080/09168451.2015.1069694 26214137

[27] Rotte C, Stejskal F, Zhu G, Keithly JS, Martin W (2001). Pyruvate : NADP^+^ oxidoreductase from the mitochondrion of *Euglena gracilis* and from the apicomplexan *Cryptosporidium parvum*: a biochemical relic linking pyruvate metabolism in mitochondriate and amitochondriate protists. Mol Biol Evol.

[28] Nakazawa M, Andoh H, Koyama K, Watanabe Y, Nakai T, Ueda M (2015). Alteration of wax ester content and composition in *Euglena gracilis* with gene silencing of 3-ketoacyl-CoA thiolase isozymes. Lipids.

[29] Teerawanichpan P, Qiu X (2010). Fatty acyl-CoA reductase and wax synthase from *Euglena gracilis* in the biosynthesis of medium-chain wax esters. Lipids.

[30] Kalscheuer R, Steinbüchel A (2003). A novel bifunctional wax ester synthase/acyl-CoA:diacylglycerol acyltransferase mediates wax ester and triacylglycerol biosynthesis in *Acinetobacter calcoaceticus* ADP1. J Biol Chem.

[31] Hoffmeister M, van der Klei A, Rotte C, van Grinsven KW, van Hellemond JJ, Henze K (2004). *Euglena gracilis* rhodoquinone:ubiquinone ratio and mitochondrial proteome differ under aerobic and anaerobic conditions. J Biol Chem.

[32] Matsuda F, Hayashi M, Kondo A (2011). Comparative profiling analysis of central metabolites in *Euglena gracilis* under various cultivation conditions. Biosci Biotechnol Biochem.

[33] Chauton MS, Winge P, Brembu T, Vadstein O, Bones AM (2013). Gene regulation of carbon fixation, storage, and utilization in the diatom *Phaeodactylum tricornutum* acclimated to light/dark cycles. Plant Physiol.

[34] Tripodi F, Nicastro R, Reghellin V, Coccetti P (1850). Post-translational modifications on yeast carbon metabolism: Regulatory mechanisms beyond transcriptional control. Biochim Biophys Acta.

[35] Koren LE, Hutner SH (1967). High-yield media for photosynthesizing *Euglena gracilis* Z. J Protozool.

[36] Arakawa K, Mori K, Ikeda K, Matsuzaki T, Kobayashi Y, Tomita M (2003). G-language genome analysis environment: a workbench for nucleotide sequence data mining. Bioinformatics.

[37] Conesa A, Götz S, García-Gómez JM, Terol J, Talón M, Robles M (2005). Blast2GO: a universal tool for annotation, visualization and analysis in functional genomics research. Bioinformatics.

[38] Kanehisa M, Goto S, Sato Y, Kawashima M, Furumichi M, Tanabe M (2014). Data, information, knowledge and principle: back to metabolism in KEGG. Nucleic Acids Res.

[39] Kono N, Arakawa K, Ogawa R, Kido N, Oshita K, Ikegami K (2009). Pathway projector: web-based zoomable pathway browser using KEGG atlas and Google Maps API. PLoS One.

[40] Langmead B, Salzberg SL (2012). Fast gapped-read alignment with Bowtie 2. Nat Methods.

[41] Robinson MD, McCarthy DJ, Smyth GK (2010). edgeR: a Bioconductor package for differential expression analysis of digital gene expression data. Bioinformatics.

[42] Li B, Dewey CN (2011). RSEM: accurate transcript quantification from RNA-Seq data with or without a reference genome. BMC Bioinformatics.

[43] Beissbarth T, Speed TP (2004). GOstat: find statistically overrepresented Gene Ontologies within a group of genes. Bioinformatics.

