# Supplementary material for: De novo assembly and comparative transcriptome analysis of Euglena gracilis in response to anaerobic conditions
Source: BMC Genomics. 2016 Mar 3;17:182. doi: 10.1186/s12864-016-2540-6 (PMC4778363; doi:10.1186/s12864-016-2540-6)
Supplement: Additional file 1: Table S1. — Summary of the homology search by BLASTX program for the selected components related to paramylon and wax ester metabolism in E.gracilis. The localization of individual deduced proteins is predicted by using TargetP 1.1 program (http://www.cbs.dtu.dk/services/TargetP/) and/or TMHMM v.2.0 (http://www.cbs.dtu.dk/services/TMHMM/). (DOCX 47 kb) [file 12864_2016_2540_MOESM1_ESM.docx]

Table S1 Summary of the homology search by BLASTX program for the selected components related to paramylon and wax ester metabolism in *E.gracilis*. The localization of individual deduced proteins is predicted by using TargetP 1.1 program (http://www.cbs.dtu.dk/services/TargetP/) and/or TMHMM v.2.0 (http://www.cbs.dtu.dk/services/TMHMM/).

| Component ID | Description | E-value | Predicted localization | FPKM | | | | | | Fold change | |
| --- | --- | --- | --- | --- | --- | --- | --- | --- | --- | --- | --- |
|  |  |  |  | 0h | SD | 12h | SD | 24h | SD | 12h/0h | 24h/0h |
| Palamylon metabolism |  |  |  |  |  |  |  |  |  |  |  |
| comp36539_c1_seq4 | Callose synthase 12 in Arabidopsis thaliana_Q9ZT82\|CALSC_ARATH | 2.00E-42 | membrane | 0.7 | 0.03 | 0.8 | 0.18 | 1 | 0.22 | 1.14 | 1.43 |
| comp36157_c0_seq1 | Callose synthase 7 in Arabidopsis thaliana_Q9SHJ3\|CALS7_ARATH | 3.00E-74 | membrane | 7.143 | 0.63 | 9.512 | 0.66 | 9.025 | 0.63 | 1.33 | 1.26 |
| comp35277_c0_seq2 | UDP-sugar pyrophospharylase in Pisum sativum_Q5W915\|USP_PEA | 5.00E-125 |  | 0.40 | 0.13 | 0.37 | 0.02 | 0.55 | 0.27 | 0.92 | 1.35 |
| comp31291_c0_seq1 | Glucan 1,3-beta-glucosidase in Candida albicans_P43070\|BGL2_CANAX | 2.00E-08 | cytosol | 19.966 | 0.45 | 25.24 | 0.94 | 24.262 | 0.78 | 1.26 | 1.22 |
| comp30671_c0_seq1 | Putative endo-1,3(4)-beta-glucanase 2 in Schizosaccharomyces pombe_Q09850\|ENG2_SCHPO | 4.00E-42 | membrane | 3.376 | 0.23 | 4.382 | 0.16 | 4.596 | 0.37 | 1.30 | 1.36 |
| comp30466_c0_seq1 | Beta-glucosidase 42 in Arabidopsis thaliana_Q9FIW4\|BGL42_ARATH | 7.00E-123 | membrane | 5 | 0.31 | 6.397 | 0.64 | 6.851 | 0.24 | 1.28 | 1.37 |
| comp24161_c0_seq2 | Beta-glucosidase in Bacillus circulans_Q9C8Y9\|BGL22_ARATH | 2.00E-62 | secretory? | 4.516 | 0.23 | 5.286 | 0.64 | 6.175 | 0.43 | 1.17 | 1.37 |
| comp24059_c0_seq2 | Lichenase in Nicotiana plumbaginifolia_P07979\|GUB_NICPL, identical to EgCel17A (Takeda et.al., 2015) | 6.00E-28 | membrane | 458.126 | 30.30 | 418.775 | 15.76 | 434.442 | 16.81 | 0.91 | 0.95 |
| comp31765_c0_seq1 | Endo-1,3(4)-beta-glucanase 1 in Schizosaccharomyces pombe_Q9UT45\|ENG1_SCHPO, identical to EgCel81A (Takeda et.al., 2015) | 1.00E-45 | membrane | 15.763 | 0.84 | 16.667 | 0.47 | 16.179 | 0.71 | 1.06 | 1.03 |
| comp30649_c0_seq1 | Endo-1,3(4)-beta-glucanase 1 in Saccharomyces cerevisiae_P53753\|ENG1_YEAST, identical to EgCel81B (Takeda et.al., 2015) | 2.00E-38 | membrane | 3.939 | 0.29 | 3.965 | 0.31 | 3.995 | 0.28 | 1.01 | 1.01 |
| Glycolysis/Gluconeogenesis | |  |  |  |  |  |  |  |  |  |  |
| comp31473_c0_seq1 | Hexokinase-1 in Homo sapiens_P19367\|HXK1_HUMAN | 6.00E-31 | cytosol | 2.541 | 0.11 | 2.332 | 0.93 | 2.486 | 0.30 | 0.92 | 0.98 |
| comp26338_c0_seq1 | Glucose-6-phosphate isomerase, cytosolic 2A in Clarkia lewisii_P29333\|G6PI2_CLALE | 0 | cytosol | 83.801 | 5.03 | 76.799 | 2.66 | 75.46 | 2.02 | 0.92 | 0.90 |
| comp25319_c0_seq1 | Glucose-6-phosphate isomerase in Drosophila melanogaster_P52029\|G6PI_DROME | 0 |  | 32.992 | 1.28 | 36.088 | 2.78 | 32.257 | 1.86 | 1.09 | 0.98 |
| comp25680_c0_seq1 | Glucose-6-phosphate isomerase in Homo sapiens_P06744\|G6PI_HUMAN | 0 |  | 30.833 | 0.85 | 32.276 | 0.67 | 29.319 | 1.35 | 1.05 | 0.95 |
| comp24300_c0_seq2 | 6-phosphofructokinase 7 in Arabidopsis thaliana_Q9C5J7\|K6PF7_ARATH | 1.00E-94 | cytosol | 29.616 | 0.39 | 26.478 | 1.43 | 24.927 | 1.83 | 0.89 | 0.84 |
| comp35213_c2_seq1 | 6-phosphofructokinase 7 in Arabidopsis thaliana_Q9C5J7\|K6PF7_ARATH | 2.00E-79 | cytosol | 0.594 | 0.42 | 0.794 | 0.29 | 1.042 | 0.17 | 1.34 | 1.75 |
| comp27950_c1_seq3 | 6-phosphofructokinase 7 in Arabidopsis thaliana_Q9C5J7\|K6PF7_ARATH | 1.00E-54 | chloroplasts | 11.638 | 1.87 | 9.88 | 1.09 | 9.957 | 1.10 | 0.85 | 0.86 |
| comp28715_c0_seq1 | Pyrophosphate--fructose-6-phosphate 1-phosphotransferase in Entamoeba histolytica_Q27651\|PPPFK_ENTHI | 2.00E-74 | chloroplasts | 2.492 | 0.48 | 2.583 | 0.34 | 3.031 | 0.36 | 1.04 | 1.22 |
| comp30567_c0_seq1 | Pyrophosphate--fructose-6-phosphate 1-phosphotransferase in Entamoeba histolytica_Q27651\|PPPFK_ENTHI | 4.00E-94 | chloroplasts | 78.719 | 3.00 | 74.907 | 1.62 | 72.693 | 2.79 | 0.95 | 0.92 |
| comp14920_c0_seq1 | Fructose-1,6-bisphosphatase, chloroplastic in Spinacia oleracea_P22418\|F16P1_SPIOL, identical to EgFBPaseI (Ogawa et.al., 2015) | 4.00E-117 | chloroplasts | 74.087 | 1.44 | 71.695 | 1.07 | 0.185 | 2.68 | 0.97 | 0.00 |
| comp28286_c0_seq1 | Fructose-1,6-bisphosphatase, chloroplastic in Pisum sativum (FBPase-II)_P46275\|F16P1_PEA, identical to EgFBPaseII (Ogawa et.al., 2015) | 1.00E-79 | chloroplasts | 0.89 | 0.19 | 1.165 | 0.20 | 1.061 | 0.28 | 1.31 | 1.19 |
| comp8127_c0_seq1 | Fructose-1,6-bisphosphatase, cytosolic in Oryza sativa subsp. japonica (FBPase-III)_Q0JHF8\|F16P2_ORYSJ, identical to EgFBPaseIII (Ogawa et.al., 2015) | 3.00E-107 | cytosol | 129.102 | 2.67 | 142.026 | 4.92 | 141.417 | 1.30 | 1.10 | 1.10 |
| comp25697_c0_seq1 | Fructose-2,6-bisphosphatase in Homo sapiens_Q16875\|F263 | 2.00E-72 |  | 4.047 | 0.62 | 4.518 | 0.62 | 4.369 | 0.20 | 1.12 | 1.08 |
| comp32961_c0_seq1 | Fructose-2,6-bisphosphatase in Rattus norvegicus_P25114\|F264_RAT | 7.00E-57 |  | 1.7 | 0.42 | 2.178 | 0.29 | 2.142 | 0.30 | 1.28 | 1.26 |
| comp19503_c0_seq1 | Fructose-2,6-bisphosphatase in Arabidopsis thaliana_Q9MB58\|F26_ARATH | 2.00E-66 |  | 3.727 | 0.15 | 4.195 | 0.52 | 4.096 | 0.33 | 1.13 | 1.10 |
| comp25660_c0_seq2 | Fructose-2,6-bisphosphatase in Arabidopsis thaliana_Q9MB58\|F26_ARATH | 2.00E-78 |  | 5.951 | 1.12 | 5.64 | 0.56 | 5.444 | 0.40 | 0.95 | 0.91 |
| comp31856_c1_seq5 | Probable 6-phosphofructo-2-kinase/fructose-2,6-bisphosphatase in Caenorhabditis elegans_Q21122\|F26_CAEEL | 2.00E-63 |  | 0.676 | 0.23 | 0.688 | 0.15 | 0.793 | 0.09 | 1.02 | 1.17 |
| comp34185_c0_seq1 | Fructose-2,6-bisphosphatase in Gallus gallus_Q91348\|F26L_CHICK | 3.00E-89 | cytosol | 2.587 | 0.45 | 3.311 | 0.45 | 2.92 | 0.36 | 1.28 | 1.13 |
| comp27161_c0_seq1 | fructose-2,6-bisphosphatase | 9.00E-10 |  | 21.538 | 1.32 | 17.421 | 1.74 | 17.605 | 0.38 | 0.81 | 0.82 |
| comp21396_c0_seq1 | Fructose-bisphosphate aldolase in Dictyostelium discoideum_Q86A67\|ALF_DICDI | 1.00E-142 |  | 1315.669 | 86.09 | 1107.371 | 83.48 | 1166.72 | 38.26 | 0.84 | 0.89 |
| comp25126_c0_seq1 | Fructose-bisphosphate aldolase in Echinococcus multilocularis_Q9GP32\|ALF_ECHMU | 3.00E-93 |  | 22.6 | 0.88 | 23.424 | 0.92 | 23.533 | 0.64 | 1.04 | 1.04 |
| comp19974_c0_seq1 | Fructose-bisphosphate aldolase B in Danio rerio_Q8JH71\|ALDOB_DANRE | 1.00E-106 |  | 30.04 | 1.73 | 25.247 | 2.79 | 27.192 | 2.43 | 0.84 | 0.91 |
| comp25642_c0_seq1 | Fructose-bisphosphate aldolase in Haemophilus influenzae_P44429\|ALF_HAEIN | 2.00E-155 |  | 393.105 | 3.06 | 371.38 | 20.19 | 356.347 | 10.11 | 0.94 | 0.91 |
| comp17468_c0_seq1 | Fructose-bisphosphate aldolase in Kluyveromyces lactis_Q9C2U0\|ALF_KLULA | 6.00E-144 |  | 1096.117 | 21.18 | 877.008 | 48.02 | 874.187 | 23.34 | 0.80 | 0.80 |
| comp20171_c0_seq1 | Triosephosphate isomerase, cytosolic in Zea mays_P12863\|TPIS_MAIZE | 1.00E-97 | cytosol | 494.134 | 32.88 | 436.543 | 31.49 | 408.709 | 12.67 | 0.88 | 0.83 |
| comp21369_c1_seq1 | Triosephosphate isomerase, cytosolic in Zea mays_P12863\|TPIS_MAIZE | 3.00E-58 | cytosol | 80.063 | 6.77 | 86.448 | 7.84 | 77.794 | 3.71 | 1.08 | 0.97 |
| comp25091_c0_seq1 | Triosephosphate isomerase, cytosolic in Zea mays_P12863\|TPIS_MAIZE | 1.00E-96 | cytosol | 84.102 | 0.62 | 76.23 | 5.49 | 75.949 | 1.85 | 0.91 | 0.90 |
| comp8148_c0_seq1 | Triosephosphate isomerase, cytosolic in Zea mays_P12863\|TPIS_MAIZE | 6.00E-108 | cytosol | 873.538 | 59.66 | 686.173 | 62.17 | 749.259 | 38.68 | 0.79 | 0.86 |
| comp36615_c0_seq1 | Glyceraldehyde-3-phosphate dehydrogenase A, chloroplastic in Spinacia oleracea_P19866\|G3PA_SPIOL | 2.00E-140 | chloroplasts | 449.036 | 18.56 | 387.204 | 3.77 | 384.648 | 8.91 | 0.86 | 0.86 |
| comp34870_c1_seq1 | NADP-dependent glyceraldehyde-3-phosphate dehydrogenase in Streptococcus equinus_Q3C1A6\|GAPN_STREI | 2.00E-53 | cytosol | 50.725 | 2.39 | 41.67 | 1.40 | 45.354 | 1.59 | 0.82 | 0.89 |
| comp23967_c1_seq1 | Glyceraldehyde-3-phosphate dehydrogenase, glycosomal in Trypanosoma cruzi_P22513\|G3PG_TRYCR | 3.00E-169 | glycosome | 364.359 | 38.70 | 340.118 | 16.21 | 332.795 | 7.67 | 0.93 | 0.91 |
| comp8156_c0_seq1 | Glyceraldehyde-3-phosphate dehydrogenase, glycosomal in Leishmania mexicana_Q27890\|G3PG_LEIME | 0 | glycosome | 5457.784 | 300.92 | 4751.967 | 128.75 | 4536.775 | 75.94 | 0.87 | 0.83 |
| comp30296_c0_seq1 | Phosphoglycerate kinase, cytosolic in Trypanosoma congolense_P41760\|PGK1_TRYCO | 2.00E-155 | cytosol | 585.799 | 24.01 | 501.312 | 8.86 | 497.068 | 4.99 | 0.86 | 0.85 |
| comp33743_c0_seq1 | Phosphoglycerate kinase in Neurospora crassa_P38667\|PGK_NEUCR | 2.00E-163 |  | 40.597 | 1.47 | 37.933 | 2.54 | 37.403 | 0.60 | 0.93 | 0.92 |
| comp30366_c1_seq1 | Phosphoglycerate kinase 2 in Homo sapiens_P07205\|PGK2_HUMAN | 0 |  | 51.888 | 8.38 | 46.889 | 4.10 | 47.938 | 4.89 | 0.90 | 0.92 |
| comp30451_c0_seq1 | Phosphoglucomutase, cytoplasmic 2 in Zea mays_P93805\|PGMC2_MAIZE | 0 | cytosol | 145.025 | 11.09 | 124.484 | 5.71 | 132.07 | 1.83 | 0.86 | 0.91 |
| comp34484_c0_seq4 | Phosphomannomutase/phosphoglucomutase in Pseudomonas syringae pv. Tomato_Q88BD4\|ALGC_PSESM | 3.00E-17 |  | 4.635 | 0.84 | 4.748 | 0.85 | 5.077 | 1.11 | 1.02 | 1.10 |
| comp33450_c1_seq3 | Phosphoglucomutase-2 in Pongo abelii_Q5RFI8\|PGM2_PONAB | 1.00E-82 |  | 2.678 | 0.08 | 3.76 | 0.50 | 3.767 | 0.51 | 1.40 | 1.41 |
| comp29409_c1_seq1 | Enolase in Treponema denticola_Q73P50\|ENO_TREDE | 0 |  | 3.057 | 0.52 | 3.615 | 0.76 | 4.217 | 0.14 | 1.18 | 1.38 |
| comp8159_c0_seq1 | Enolase in Treponema denticola_Q73P50\|ENO_TREDE | 0 |  | 1468.273 | 102.07 | 1334.618 | 53.41 | 1268.304 | 18.53 | 0.91 | 0.86 |
| comp23081_c0_seq1 | Enolase in Treponema pallidum subsp. Pallidum_B2S455\|ENO_TREPS | 0 |  | 519.992 | 27.77 | 458.702 | 13.45 | 459.248 | 6.06 | 0.88 | 0.88 |
| comp31136_c1_seq3 | Pyruvate kinase PKLR in Rattus norvegicus_P12928\|KPYR_RAT | 2.00E-162 | cytosol | 49.443 | 3.33 | 54.801 | 2.28 | 52.971 | 3.25 | 1.11 | 1.07 |
| Fatty acid metabolism |  |  |  |  |  |  |  |  |  |  |  |
| comp12747_c0_seq1 | Pyruvate dehydrogenase [NADP(+)], mitochondrial in Euglena gracilis_Q94IN5\|PNO_EUGGR | 0 | mitochondria | 450.687 | 30.12 | 275.511 | 8.93 | 268.599 | 17.79 | 0.61 | 0.60 |
| comp26715_c0_seq4 | Probable pyruvate dehydrogenase E1 component subunit alpha, mitochondrial in Caenorhabditis elegans_P52899\|ODPA_CAEEL | 4.00E-121 | mitochondria | 41.26 | 4.52 | 43.406 | 3.85 | 41.437 | 2.43 | 1.05 | 1.00 |
| comp21099_c0_seq1 | Pyruvate dehydrogenase E1 component subunit beta-1, mitochondrial in Arabidopsis thaliana_Q38799\|ODPB1_ARATH | 2.00E-151 | mitochondria | 47.821 | 6.21 | 46.752 | 3.14 | 45.531 | 4.04 | 0.98 | 0.95 |
| comp27519_c0_seq1 | 2-oxoisovalerate dehydrogenase subunit beta 2, mitochondrial in Arabidopsis thaliana_Q9LDY2\|ODBB2_ARATH | 3.00E-126 | mitochondria | 17.462 | 1.61 | 16.785 | 1.86 | 16.018 | 1.35 | 0.96 | 0.92 |
| comp29498_c0_seq1 | Lipoamide acyltransferase component of branched-chain alpha-keto acid dehydrogenase complex, mitochondrial in Caenorhabditis elegans_Q23571\|ODB2_CAEEL | 2.00E-69 | mitochondria | 9.103 | 1.51 | 9.299 | 0.96 | 9.392 | 1.41 | 1.02 | 1.03 |
| comp31034_c0_seq1 | Dihydrolipoyl dehydrogenase in Trypanosoma cruzi_P90597\|DLDH_TRYCR | 3.00E-180 | mitochondria | 56.722 | 2.11 | 57.302 | 4.44 | 57.565 | 0.18 | 1.01 | 1.01 |
| comp36638_c0_seq1 | Acetyl-CoA acetyltransferase in Clostridium difficile_Q18AR0\|THLA_CLOD6_reported as 3-ket acyl-CoA thiolase (EgKAT1) | 9.00E-109 | mitochondria | 60.323 | 29.52 | 60.116 | 10.99 | 55.287 | 14.28 | 1.00 | 0.92 |
| comp17496_c0_seq1 | Acetyl-CoA acetyltransferase in Clostridium difficile_Q18AR0\|THLA_CLOD6_reported as 3-ket acyl-CoA thiolase (EgKAT2) | 3.00E-93 | mitochondria | 541.521 | 31.93 | 455.193 | 2.76 | 427.262 | 6.27 | 0.84 | 0.79 |
| comp17821_c0_seq1 | Acetyl-CoA acetyltransferase in Clostridium difficile_Q18AR0\|THLA_CLOD6_reported as 3-ket acyl-CoA thiolase (EgKAT3) | 1.00E-97 | cytosol? | 1743.882 | 41.38 | 1343.294 | 100.05 | 1284.476 | 28.11 | 0.77 | 0.74 |
| comp31551_c0_seq1 | Acetyl-CoA acetyltransferase in Clostridium difficile_Q18AR0\|THLA_CLOD6_reported as 3-ket acyl-CoA thiolase (EgKAT4) | 3.00E-99 | mitochondria | 693.936 | 5.02 | 556.448 | 3.31 | 534.237 | 1.79 | 0.80 | 0.77 |
| comp33055_c0_seq1 | 3-ketoacyl-CoA thiolase A, peroxisomal in Rattus norvegicus_P21775\|THIKA_RAT__reported as EgKAT5 | 7.00E-163 | peroxisome | 47.414 | 3.00 | 46.91 | 2.67 | 43.972 | 2.78 | 0.99 | 0.93 |
| comp22846_c0_seq1 | Probable acetyl-CoA acetyltransferase, cytosolic 2 in Arabidopsis thaliana_Q9FIK7\|THIC2_ARATH_reported as 3-ket acyl-CoA thiolase (EgKAT6) | 3.00E-130 | cytosol | 24.888 | 3.02 | 28.855 | 2.08 | 28.315 | 0.66 | 1.16 | 1.14 |
| comp24971_c0_seq1 | 3-hydroxyacyl-CoA dehydrogenase type-2 in Bos taurus_O02691\|HCD2_BOVIN | 4.00E-77 | mitochondria | 49.978 | 2.13 | 44.668 | 1.57 | 47.988 | 2.00 | 0.89 | 0.96 |
| comp25049_c0_seq1 | Hydroxyacyl-coenzyme A dehydrogenase, mitochondrial in Rattus norvegicus | 6.00E-66 | mitochondria | 2812.192 | 120.86 | 2153.396 | 152.20 | 1967.768 | 109.35 | 0.77 | 0.70 |
| comp36568_c0_seq1 | Probable 3-hydroxyacyl-CoA dehydrogenase F54C8.1 in Caenorhabditis elegans | 3.00E-64 | mitochondria | 3064.848 | 108.98 | 2318.87 | 50.22 | 2171.768 | 137.48 | 0.76 | 0.71 |
| comp29182_c0_seq3 | Delta(3,5)-Delta(2,4)-dienoyl-CoA isomerase, mitochondrial in Dictyostelium discoideum_Q54SS0\|ECH1_DICDI | 9.00E-70 | mitochondria | 14.706 | 0.89 | 14.649 | 1.70 | 16.007 | 0.35 | 1.00 | 1.09 |
| comp20039_c0_seq1 | Probable enoyl-CoA hydratase echA8 in Mycobacterium tuberculosis_P64016\|ECHA8_MYCTU | 5.00E-98 | mitochondria | 564.661 | 13.46 | 466.436 | 11.49 | 483.203 | 18.43 | 0.83 | 0.86 |
| comp34527_c0_seq1 | *Trans*-2-enoyl-CoA reductase, mitochondrial in Euglena gracilis (TER1)_Q5EU90\|TER_EUGGR | 0 | plastid or mitochondria | 15.496 | 0.72 | 15.499 | 1.98 | 16.871 | 1.38 | 1.00 | 1.09 |
| comp27719_c0_seq1 | *Trans*-2-enoyl-CoA reductase, mitochondrial in Dictyostelium discoideum (TER2)_Q54YT4\|MECR_DICDI | 1.00E-33 | mitochondria | 113.719 | 1.56 | 107.838 | 4.08 | 102.969 | 2.66 | 0.95 | 0.91 |
| comp34564_c0_seq6 | Short-chain specific acyl-CoA dehydrogenase, mitochondrial in Rattus norvegicus_P15651\|ACADS_RAT | 4.00E-167 |  | 47.58 | 4.61 | 47.914 | 2.59 | 44.66 | 2.13 | 1.01 | 0.94 |
| comp32935_c0_seq1 | Isobutyryl-CoA dehydrogenase, mitochondrial in Dictyostelium discoideum_Q54IM8\|ACAD8_DICDI | 5.00E-162 |  | 36.09 | 0.66 | 39.771 | 3.08 | 33.397 | 0.21 | 1.10 | 0.93 |
| comp29154_c0_seq1 | Acyl-CoA dehydrogenase in Bacillus subtilis_P45857\|ACDB_BACSU | 2.00E-32 |  | 1261.535 | 65.39 | 844.702 | 21.61 | 804.904 | 66.94 | 0.67 | 0.64 |
| Wax ester metabolism |  |  |  |  |  |  |  |  |  |  |  |
| comp32985_c0_seq2 | Fatty acyl-CoA reductase 1 in Gallus gallus_Q5ZM72\|FACR1_CHICK, identical to Euglena acyl-CoA reductase | 1.00E-63 |  | 5.893 | 0.46 | 6.16 | 0.61 | 6.665 | 0.24 | 1.05 | 1.13 |
| comp25371_c1_seq1 | Probable long-chain-alcohol O-fatty-acyltransferase 3 in Arabidopsis thaliana_Q9FJ74\|WAXS3_ARATH, identical to Euglena wax ester syntase (WS1) | 4.00E-13 |  | 1.414 | 0.46 | 1.182 | 0.53 | 1.289 | 0.13 | 0.84 | 0.91 |
| comp17081_c0_seq1 | Acyl-CoA--sterol O-acyltransferase 1 in Arabidopsis thaliana_Q9SV07\|ASAT1_ARATH | 5.00E-11 |  | 6.246 | 0.45 | 5.247 | 0.16 | 4.753 | 0.15 | 0.84 | 0.76 |
| comp29378_c0_seq1 | Putative diacyglycerol O-acyltransferase Mb3154c in Mycobacterium bovis_P0A651\|Y3154_MYCBO | 2.00E-14 |  | 2.953 | 0.65 | 2.262 | 0.29 | 2.871 | 0.50 | 0.77 | 0.97 |
| comp17367_c0_seq1 | Putative diacyglycerol O-acyltransferase Mb0919 in Mycobacterium bovis_P67205\|Y919_MYCBO | 3.00E-14 |  | 41.802 | 0.88 | 35.502 | 1.50 | 36.134 | 1.02 | 0.85 | 0.86 |
| comp31582_c0_seq2 | Putative diacyglycerol O-acyltransferase Rv1425/MT1468 in Mycobacterium tuberculosis_P71694\|Y1425_MYCTU | 1.30E-14 |  | 23.037 | 1.51 | 22.934 | 1.43 | 21.659 | 1.96 | 1.00 | 0.94 |
| comp34975_c0_seq2 | Putative diacyglycerol O-acyltransferase Rv1760/MT1809 in Mycobacterium tuberculosis_O06795\|Y1760_MYCTU | 1.50E-07 | cytosol | 6.14 | 0.29 | 6.712 | 0.68 | 7.021 | 0.65 | 1.09 | 1.14 |
| comp33545_c0_seq1 | Putative diacyglycerol O-acyltransferase Rv1760/MT1809 in Mycobacterium tuberculosis_O06795\|Y1760_MYCTU | 3.00E-11 | cytosol | 25.105 | 0.58 | 26.502 | 1.00 | 23.549 | 0.96 | 1.06 | 0.94 |
| comp34299_c0_seq1 | Putative diacyglycerol O-acyltransferase Rv1760/MT1809 in Mycobacterium tuberculosis_O06795\|Y1760_MYCTU | 4.00E-37 | cytosol | 7.984 | 0.56 | 6.055 | 0.12 | 5.757 | 0.36 | 0.76 | 0.72 |
