# Supplementary material for: De novo assembly and comparative transcriptome analysis of Euglena gracilis in response to anaerobic conditions
Source: BMC Genomics. 2016 Mar 3;17:182. doi: 10.1186/s12864-016-2540-6 (PMC4778363; doi:10.1186/s12864-016-2540-6)
Supplement: Additional file 2: Table S2. — Summary of reads for sequencing of individual cDNA libraries constructed from E.gracilis treated with aerobic- and anaerobic conditions. (DOCX 18 kb) [file 12864_2016_2540_MOESM2_ESM.docx]

Table S2 Summary of reads for sequencing of individual cDNA libraries constructed from *E.gracilis* treated with aerobic- and anaerobic conditions.
