# Supplementary figures and images for: De novo assembly and comparative transcriptome analysis of Euglena gracilis in response to anaerobic conditions
Source: BMC Genomics. 2016 Mar 3;17:182. doi: 10.1186/s12864-016-2540-6 (PMC4778363; doi:10.1186/s12864-016-2540-6)

## Slide 1
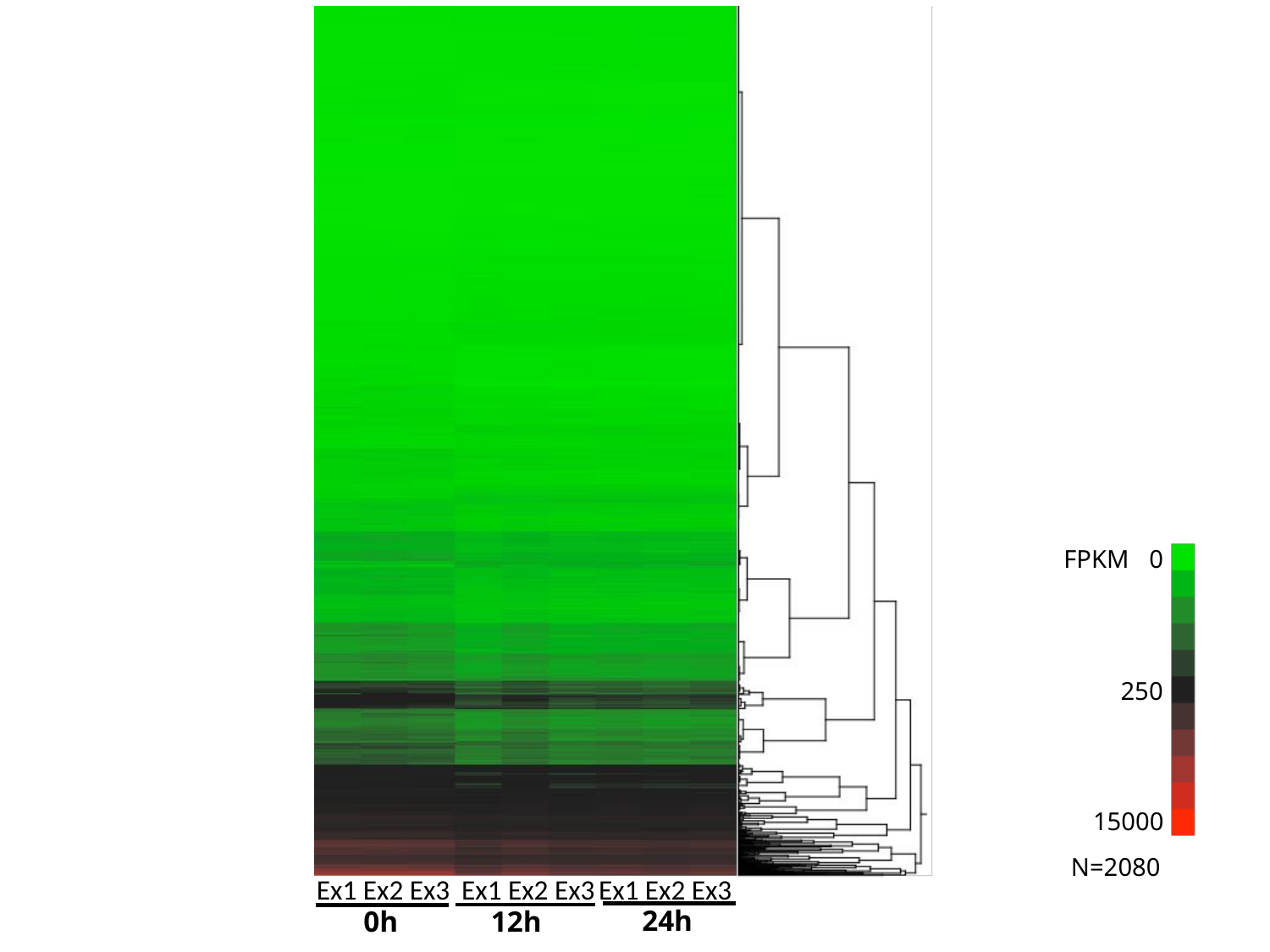

FPKM
0
250
15000
N=2080
Ex1 Ex2 Ex3
Ex1 Ex2 Ex3
Ex1 Ex2 Ex3
24h
0h
12h

Supplement: Additional file 4: Figure S1. — Hierarchical clustering analysis for 2080 DEGs in response to anaerobic conditions. Each row and column represent individual DEGs and time points, respectively. For each time point, the experiment was performed three times each. (PPTX 91 kb) [file 12864_2016_2540_MOESM4_ESM.pptx]
